# Training biologists in Unix command-line skills: From curriculum to interactive online tutorials

**DOI:** 10.1371/journal.pcbi.1014133

**Published:** 2026-04-03

**Authors:** Lucie Khamvongsa-Charbonnier, Robert Aboukhalil, Hélène Chiapello, Thomas Denecker, Pierre Poulain, Denis Puthier, Olivier Sand, Morgane Thomas-Chollier, Claire Toffano-Nioche

**Affiliations:** 1 IFB-core, Institut Français de Bioinformatique (IFB), CNRS, INSERM, INRAE, CEA, Villejuif, France; 2 OMGenomics Labs, San Francisco, California, United States of America; 3 Université Paris-Saclay, INRAE, MaIAGE, Jouy-en-Josas, France; 4 Université Paris Cité, CNRS, Laboratoire de Biochimie Théorique, Paris, France; 5 Aix-Marseille Université, INSERM, TAGC, TGML, MarMaRa Institute, Turing Centre for Living systems, Transcriptomics and Genomics Marseille Luminy (TGML), Marseille, France; 6 Institut de biologie de l’ENS (IBENS), École normale supérieure, CNRS, INSERM, Université PSL, Paris, France; 7 Université Paris-Saclay, CEA, CNRS, Institute for Integrative Biology of the Cell (I2BC), Gif-sur-Yvette, France; SIB: Swiss Institute of Bioinformatics, SWITZERLAND

## Abstract

As the generation of data in the life and health sciences expands rapidly, there is a growing need for professionals and students in these fields to master core bioinformatics skills, particularly those relating to Unix-like systems, most commonly used in bioinformatics. This paper introduces two key contributions to address this need: (1) A Unix curriculum for life scientists with little or no command-line experience, based on progressive Unix skill levels for bioinformatics and (2) An implementation of this curriculum into a series of interactive online tutorials deployed through Sandbox.bio—an open-source platform for learning bioinformatics that embeds a command line in the browser, which removes barriers related to software installation and access. We performed an overall evaluation of this teaching framework in different contexts. This inclusive, sustainable approach provides widespread access to essential bioinformatics skills for life science students and professionals alike.

## Introduction

As data generation continues to accelerate, the life science and health communities are facing a growing need to master core bioinformatics skills. These communities encompass a diverse range of profiles from university undergraduates to professionals in research and medical institutions, eager to learn new analysis techniques [1–4]. Training such a large and diverse community raises significant challenges, especially regarding content design, trainer availability, and access to computational resources [5,6]. The International Society for Computational Biology Education committee has recently proposed with international experts a competency framework for bioinformatics including knowledge, skills, and attitudes (KSAs) for several bioinformatics career profiles [7]. In this context, Unix, Python, and R are commonly identified as core technical skills in data science in general and also in many bioinformatics training programs [8,9]. One notable example is the Software Carpentry initiative [10], which has been playing a key role in providing high-quality training and open material to the community (see: https://software-carpentry.org). However, this initiative largely depends on face-to-face training, which creates a bottleneck due to the need for a sufficient number of trained instructors. It also requires using a commercial cloud (Amazon Web Services) or installing several bioinformatics tools locally (using the command-line interface!).

In recent years, self-paced learning has emerged as a key pillar of education, offering flexible, learner-centered approaches that allow individuals to progress at their own pace, manage their schedules, and revisit foundational concepts as needed [11]. This format is particularly well-suited for acquiring prerequisites ahead of in-person courses, managing large student cohorts, and supporting lifelong learning in professional environments. To meet this demand, several platforms—such as DataCamp (https://www.datacamp.com], Katacoda [now closed], and Killercoda (https://killercoda.com)—have contributed to democratizing self-paced learning in fields like data science and programming. However, these solutions remain proprietary, raising concerns about long-term availability (e.g., Katacoda was discontinued in 2022 [12]), and are generally not designed for bioinformatics. More broadly, many free and open educational resources have been developed to support training in computational biology. For instance, the Galaxy Training Network [13,14] is a prominent collaborative platform offering open-source tutorials tailored for both scientists and trainers across a wide range of topics. TeSS, the ELIXIR Training eSupport System, has a dedicated section to discover existing e-learning materials (https://tess.elixir-europe.org/elearning_materials). Additionally, an increasing number of bioinformatics tools now come with dedicated tutorials for educators and beginners, facilitated by the accessibility and collaborative features of platforms like GitHub and GitLab. Yet, they often assume access to sufficient computing infrastructure—whether local machines or remote clusters—and a minimum level of system configuration skills. These technical requirements pose a significant barrier for learners with limited prior experience, especially in institutional settings and countries where access to resources is uneven.

In this context, WebAssembly (Wasm) technologies offer a transformative alternative by enabling programs to run directly within the user’s browser. This approach harnesses the user’s own computing power, eliminating the reliance on large centralized computational infrastructures. Moreover, software installation and local configuration are handled seamlessly, enabling effortless deployment and a smooth user experience. As most introductory tutorials make use of small datasets for demonstration purposes, a basic web browser serving basic files for a website (e.g., HTML, CSS, Javascript) together with Wasm-compiled programs should be generally sufficient for training. Wasm thus appears as a promising solution to meet the needs of online training for bioinformatics core skills where complex toolchains and system dependencies often hinder early learning. For instance, JupyterLite (https://jupyterlite.readthedocs.io/en/latest/), a Wasm implementation of JupyterLab, allows running a fully-fledged JupyterLab in the browser without the need of any external server.

Relying on the WebAssembly technology, Sandbox.bio (https://sandbox.bio) represents a forward-looking step in bioinformatics education. At the initiative of one of the authors of this article (RA), it has now become an open-source online platform specifically designed for bioinformatics training. This platform leverages the use of the Wasm technology to provide self-paced guided tutorials for popular bioinformatics programs such as BLAST, SeqKit, or fastp. Sandbox.bio also provides virtual playgrounds where learners can experiment with Unix tools such as Grep, Awk, or Sed.

Despite the consensus that Unix is a fundamental skill in bioinformatics, the specific commands and levels required in the field of bioinformatics are rarely defined beyond a generic level like “basic,” “intermediate,” or “advanced,” generally not attached to any precise operational skills. For example, does a biology student learning Unix need to know all or only some of the Unix commands related to data manipulation (like, grep, cut, count, awk…)? Does he/she need to learn to write a complete Bash script or rather only need to understand how a given script is working and be able to adapt it to his/her needs? It has become crucial to go beyond generic levels to truly unlock self-paced learning, and globally facilitate learning of Unix skills for the life sciences.

In this work, we address two issues: Firstly, we designed a Unix curriculum targeting life scientists with little or no command line experience and defined progressive Unix skill levels in bioinformatics that go beyond the three generic basic, intermediate, and advanced levels. Secondly, we propose sustainable online tutorials to help biologists master these skills. The Unix curriculum details and organizes each individual Unix concept in coherent and progressive categories. This curriculum can be used for various purposes, such as self-assessing student level, designing a training curriculum based on learner profile, or specifying target skills to be acquired upon completion of the training. Secondly, we propose a series of interactive online tutorials based on the WebAssembly technology and hosted on the open-source sandbox.bio platform. This enables access without installation and supports a large number of trainees.

These two resources can be used independently, yet they were designed to be complementary, with the tutorials directly covering the topics outlined in the skill levels. Designed for biologists, these resources enable the progressive learning of bioinformatics with biology-specific examples. Hosted by the sandbox.bio platform, they are inexpensive in terms of computing resources and can be deployed on a large scale without posing any beginner-related risk to computing infrastructures, such as breaking something on shared servers.

## Methodology

### A curriculum including five Unix skill levels

We used the tips described in [15] to develop a Unix command line curriculum for life scientists with little or no command line experience. First, we checked that the training need was unmet and no similar training material was available elsewhere (i.e., Unix command line bioinformatics, tips 1 & 2). Second, we ensured a balance of conceptual and practical exercises (tip 3), explained terminology (tip 5), and customized data files relevant for Life Scientist Audience (tip 7). We also collected feedback and revised some parts (tip 8, see below “Curriculum evaluation” section) and made training materials openly accessible for trainees and trainers (tip 9).

To design this curriculum, we first described Unix-relevant skills for bioinformatics within the framework of the revised Bloom cognitive taxonomy framework [16]. Bloom’s taxonomy classifies educational objectives into six different categories: Remember, Understand, Apply, Analyze, Evaluate, and Create. We then used these categories to compile a list of increasing complexity abilities within multiple skill levels. These abilities were also grouped into themes, ranging from understanding what a command-line interface is to running a genomics analysis software in the terminal.

### Tutorial implementation

Based on the Unix skill levels, we created step-by-step tutorials to guide learners. Each tutorial consists of a couple of steps interspersed with quizzes, and covers at least one of the categories from Bloom’s taxonomy. The tutorials were written in Markdown format and parametrized with a JSON file.

### Tutorial deployment

The Markdown source files for the tutorials are freely available in a GitHub repository (https://github.com/sandbox-bio/sandbox.bio). The tutorials were deployed on a virtual machine running the Sandbox.bio platform (https://sandboxbio.france-bioinformatique.fr). Sandbox.bio is an open-source platform that offers command-line emulation based on the v86 project (https://github.com/copy/v86), a Wasm-compiled x86 emulator that runs in the browser. Its source code is freely available at https://github.com/sandbox-bio/sandbox.bio and archived in Software Heritage (swh:1:dir:e88a9a270b2e318350d741308797c55fc07bff48). We built the Docker image that powers the command-line interface on an Ubuntu 22.04.5 LTS virtual machine with 8 GB of RAM and 2 CPUs, and Node.js was used as the web server. We adapted the original Sandbox.bio code so that the folder organization mimics the one of the ELIXIR-FR/IFB high-performance computing (HPC) infrastructure. This ensures that users learning on these tutorials are well-prepared for moving afterwards on the HPC infrastructure.

Once deployed, each tutorial is accessible as a “Unix module” on a public web page hosted by the French Institute of Bioinformatics (ELIXIR-FR/IFB), which is part of the European Elixir infrastructure.

## Results and discussion

### Curriculum for learning bioinformatics

**Fig 1:**
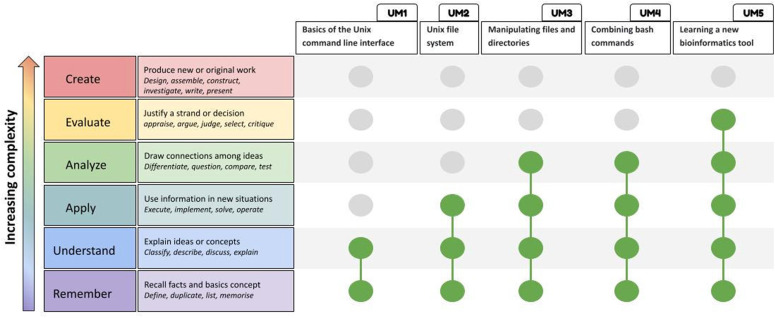
Application of Bloom’s revised taxonomy activities to design Unix skills of increasing difficulty. The left-hand side of the figure shows the revised Bloom’s taxonomy. The columns at the top describe the groups of Unix skills that are mandatory for bioinformatics, classified by theme and ordered by increasing difficulty. These groups are named UM 1 to UM 5, in reference to the Unix Modules developed in the sandbox.bio tutorials.

**Fig 2:**
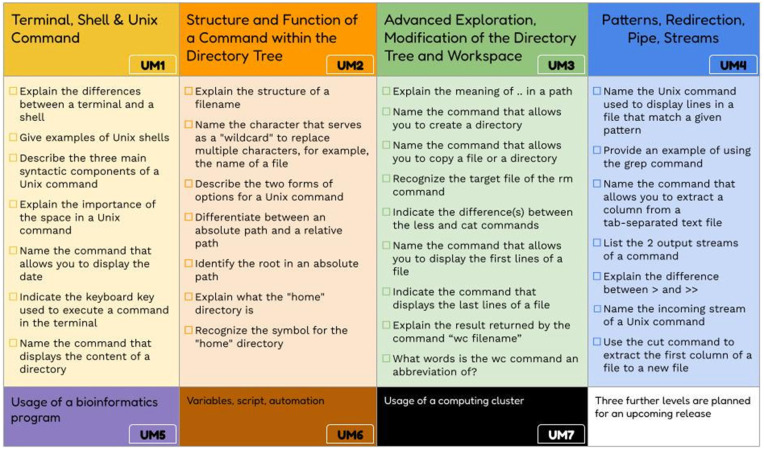
The four skill levels and learning outcomes for bioinformaticians (U1 to U4). The table presents examples of learning outcomes for each level; refer to the complete documents to view the full extent [15,16]. U5 to U7 refers to three additional levels that will be defined in future versions.

We defined five Unix skill levels and used Bloom’s taxonomy to classify the required skills (see Fig 1). The level of difficulty increases, with the skills becoming cumulative: those acquired at one level are necessary to reach the next. The five levels (see Table 1 and Fig 2) are named UM (for Unix Module) and cover the following topics: UM1 (terminal, shell and Unix commands), UM2 (structure and operation of a command in the file system), UM3 (advanced exploration of the file system, and workspace), and UM4 (motifs, redirection, pipe, flux), UM5 (learning a new bioinformatics tool and manage errors). Table 1 and Fig 2 include examples of the learning outcomes for each level. The full skills sheet is freely available in Zenodo in French [17] and in English [18].

**Table 1:** A Unix curriculum for training life scientists to the command line.

**Target audience**	**Life scientists with no or minimal command-line experience, working with sequencing or other omics data**
**Format**	5 online modules, each 1 to 1.5 hours, mixing guidance and hands-on exercises on a realistic biological dataset
**Unix Module 1 (UM1) – Basics of the Unix command line interface**	**Outcomes:** understand what is a terminal and a shell, the Unix command structure, open a terminal, run a simple command, get basic help **Topics:** shell, terminal, command (name, argument), date, ls, wildcard, file, directory, man
**Unix Module 2 (UM2) – Unix file system**	**Outcomes:** understand the Unix file system (tree, paths), create directories, copy/move/delete files **Topics:** pwd, tree, absolute and relative paths, cd, automatic completion, HOME directory, mkdir, cp, mv, rm
**Unix Module 3 (UM3) – Manipulating files and directories**	**Outcomes:** manipulate file content and data **Topics:** cat, less, head, tail, gzip, unzip, wc, grep, cut, history
**Unix Module 4 (UM4) – Combining bash commands**	**Outcomes:** chain several commands using redirections and pipes **Topics:** standard output, input and error, > , pipe, sort, echo
**Unix Module 5 (UM5) – Discovering a new bioinformatics tool**	**Outcomes:** explore how to use a command-line bioinformatics tool (example with SeqKit) **Topics:** get documentation using man and help, look at version using which, look at arguments and options, manage inputs, outputs, and frequent errors

### Online tutorials for practical, self-paced, and autonomous learning of Unix

We used the five Unix skill levels to design five Unix tutorials. The content of the tutorials is available in HAL, a French public open science repository (https://hal.science/hal-05506951). The skill levels structure the curriculum, while the interactive tutorials provide, hands-on exercises.

The tutorials are tagged and versioned. At the time of writing this article, the current release is v2.0. (https://github.com/IFB-ElixirFr/sandboxbioscenarios/releases/tag/v2.0-hal-02-2026/) and distributed through a dedicated Sandbox.bio instance at the French Institute of Bioinformatics (ELIXIR-FR/IFB): https://sandboxbio.france-bioinformatique.fr/. Each tutorial includes pedagogical support, with explanations of concepts and commands on the left, and a Linux terminal for running Unix commands in a secure environment on the right side.

### Curriculum evaluation

The implemented curriculum has been tested in multiple educational settings:

- In autonomy pre-class activities for professional bioinformatics training: “Introduction to the processing of genomic data obtained by high-throughput sequencing” at the French Bioinformatics Institute (EB3I 2023,2024, 2025: 40 trainees in each session), a one-week intensive training session for life scientists.
- In autonomy pre-class activities for a professional diploma at Université Paris Cité: “Production, analysis, and valorization of biological omics data” (DU omiques 2023 and DU omiques 2025, with 14 trainees in each session).
- In autonomy pre-class activities for biologists and interns (14 participants) at the Institute for Integrative Biology of the Cell (I2BC) at Université Paris-Saclay
- Self-assessment of Linux skills by 30 students of the IMALIS life science master’s program at the École Normale Supérieure (Paris).
- Self-assessment of Linux skills by 40 students of the Polytech graduate school of engineering (specialty in Biological Engineering) at Aix-Marseille University.

We assessed the perception of these online tutorials through a survey after the 2023 EB3I training: 90% of participants found the tutorials clear, and 95% considered them to be of an appropriate length. In the EB3I 2024 edition, these figures increased to 97% and 92%, respectively, indicating overall positive feedback of the tutorials. Participants quoted: “I loved it, very instructive.”, “Very practical, it allows me to learn about the command line and Bash.”, “[This online training] is essential because it lays the necessary foundations for the week. I found this pre-training very appropriate and well-guided, with the option of typing command lines in parallel.” Many trainees also reported that a key advantage of these tutorials was that they combine step-by-step instructions together with an interactive terminal providing a safe and integrated environment for trainees to progress at their own pace.

These positive feedbacks highlight that these tutorials are suited for students and professionals alike. By providing ready-to-use yet realistic environments, these tutorials bring a first step toward the use of more complex and powerful computing environments. For example, we are considering a tutorial to help users access the ELIXIR-FR/IFB high-performance computing cluster.

Interestingly, our work meets several of the grand challenges in bioinformatics education and training recently pointed out by Işık and colleagues [5]: GC1: Identifying fundamental knowledge and skills, GC2: supporting lifelong training, GC6: Practicing inclusivity and equity in bioinformatics education and GC7: Ensuring the sustainability of bioinformatics education.

At this stage, the developed training resources are limited to the basic Unix core skills required for using bioinformatics tools. While this alone does not provide a full bioinformatics training, it serves as an essential first step toward becoming a bioinformatics user, as defined in [6]. This approach meets a significant demand within the Life Sciences, Agronomy, and Medical Research communities. Moreover, additional tutorials and resources are available for those wishing to further develop their Unix skills. For instance, the sandbox.bio platform offers additional tutorials, such as “*How to write a bash script”* and “*Data exploration with awk*” (available at https://sandbox.bio/tutorials). At I2BC-University Paris-Saclay, GameShell (https://github.com/phyver/GameShell) was used to go further in the use of the Unix command line. Although this tool requires local installation, it guides participants through Unix commands with a dungeon discovery adventure. Going forward, we intend to continue developing free online tutorials and platforms to promote the large-scale development of bioinformatics skills.
