# Correction: Training biologists in Unix command-line skills: From curriculum to interactive online tutorials

**DOI:** 10.1371/journal.pcbi.1014645

**Published:** 2026-08-11

**Authors:** Lucie Khamvongsa-Charbonnier, Robert Aboukhalil, Hélène Chiapello, Thomas Denecker, Pierre Poulain, Denis Puthier, Olivier Sand, Morgane Thomas-Chollier, Claire Toffano-Nioche

All authors should be noted as contributing equally to this work.

## References

[1] Khamvongsa-Charbonnier L, Aboukhalil R, Chiapello H, Denecker T, Poulain P, Puthier D, et al. Training biologists in Unix command-line skills: From curriculum to interactive online tutorials. PLoS Comput Biol. 2026;22(4):e1014133. doi: 10.1371/journal.pcbi.1014133 41931477 PMC13048368

